# Polarization insensitivity characterization of dual-band perfect metamaterial absorber for K band sensing applications

**DOI:** 10.1038/s41598-021-97395-0

**Published:** 2021-09-08

**Authors:** Mohammad Lutful Hakim, Touhidul Alam, Ali F. Almutairi, Mohd Fais Mansor, Mohammad Tariqul Islam

**Affiliations:** 1 Pusat Sains Angkasa (ANGKASA), Institut Perubahan Iklim, Universiti Kebangsaan Malaysia, 43600 UKM, Bangi, Selangor Malaysia; 2 Department of CSE, International Islamic University Chittagong, Kumira, Chittagong 4318 Bangladesh; 3 Electrical Engineering Department, Kuwait University, 13060 Kuwait City, Kuwait; 4 Department of Electrical, Electronic and Systems Engineering, Faculty of Engineering and Built Environment, Universiti Kebangsaan Malaysia, 43600 Bangi, Malaysia

**Keywords:** Electrical and electronic engineering, Electronic structure

## Abstract

Polarization insensitive metamaterial absorbers (MA) are currently very attractive due to their unique absorption properties at different polarization angles. As a result, this type of absorber is widely used in sensing, imaging, energy harvesting, etc. This paper presents the design and characterization of a dual-band polarization-insensitive metamaterial absorber (MA) for K-band applications. The metamaterial absorber consists of two modified split ring resonators with an inner cross conductor to achieve a 90% absorption bandwidth of 400 MHz (21.4–21.8 GHz) and 760 MHz (23.84–24.24 GHz) at transverse electromagnetic (TEM), transverse electric (TE), and transverse magnetic (TM) mode. Polarization insensitivity of different incident angles for TE and TM mode is also investigated, which reveals a similar absorption behavior up to 90°. The metamaterial structure generates single negative (SNG) property at a lower frequency of 21.6 GHz and double negative property (DNG) at an upper frequency of 24.04 GHz. The permittivity and pressure sensor application are investigated for the proposed absorber, which shows its useability in these applications. Finally, a comparison with recent works is also performed to demonstrate the feasibility of the proposed structure for K band application, like sensor, filter, invasive clock, etc.

## Introduction

Metamaterial is an artificial structure that consolidates startling characteristics like negative permeability and/or permittivity, which leads to a negative or positive refractive index and backward propagation^1^. This special phenomenon facilitates researchers to use metamaterial in various fields like absorption, sensing, antenna design, mobile applications, military purposes, reduction of radar cross-section, electromagnetic clock, filter, waveguide, lenses, etc^2–10^. Metamaterial perfect absorber (MPA) was first introduced by Landy et al.^11^, which attracted great interest because it absorbs almost all incidents of electromagnetic wave, and very negligible or nothing is reflected back from the absorber surface, which leads to different uses like energy harvesting^12^, sensing^2^, optical^13^, switching^14^, undesired frequency absorption^15^, etc. Polarization insensitivity is the essential feature of MPA because the position of absorption bandwidth and absorption level remains unchanged at different polarization angles. This feature is achieved by a high degree of symmetrical structure design^16,17^. Normally a three-layer MPA is designed, in which patch and ground are separated by a substrate material that generates coupling capacitance. Two layer^18^ multilayers ^19^ are also used for MPA design. MPA is designed for Hz, GHz, and THz applications, wherein for GHz use researchers work on designing C, X, Ku, and K band applications^20^. Three-layer symmetrical structured square split ring resonator^21^ and concentric close circular ring resonator^22^ for C, X, and Ku band metamaterial absorber is designed, in which polarization incident angle stability is 60° and 75°, respectively. 60° and 70° polarization insensitivity was achieved for Ku band application by Jerusalem cross^23^ and a shorted stub circular ring (CR)^24^ three-layer symmetrical structure. Many researchers are working on X and Ku band applications with three layer symmetrical structure with different patch designs like ring C shape ^25^, Jerusalem crosses with square ring resonator (SRR)^26^, cross shape resonator (CSR), and complimentary cross shape resonator CCSR^27^. All three researchers achieved polarization insensitivity up to 60°. In^28^ a small size circular ring resonator is designed for X and Ku bands, wherein polarization insensitivity is only 15°. 90° polarization-insensitive is achieved by a three-layer symmetrical MPA for X, Ku, and K band application in^29^. For Ku and K bands a spiral shape absorber atom is designed for 80° polarization angle insensitivity^30^. A quarter spit ring with inner steric resonator for K band was introduced by the author^31^, where 85° polarization insensitivity is realized. Polarization sensitivity and lower absorption level are the major challenges of MPA design. Metamaterial absorber with wide polarization insensitivity for K band application is rarely found. K band is preferable for short range absorption and sensing applications because of the high attenuation at this band. This paper takes initiative for designing a K band metamaterial absorber with width polarization angle insensitivity. A symmetric structured modified circular split ring resonator (CSRR) with an inner cross is proposed for K band application. The structure shows 90° polarization insensitivity for both normal and oblique incident EM wave at TEM mode and realized 99.9% of absorption at 21.6 and 24.04 GHz. This excellent absorption behavior makes the proposed a perfect metamaterial absorber.

The remainder of the paper is arranged in such a way that design and simulation setup are elaborated in Sect. 2. Results and discussion are presented in Sect. 3, sensor performance is investigated in Sect. 4, and a comparison of the proposed MPA with similar work is tabulated in Sect. 5.

## Design and simulation

**Figure 1:**
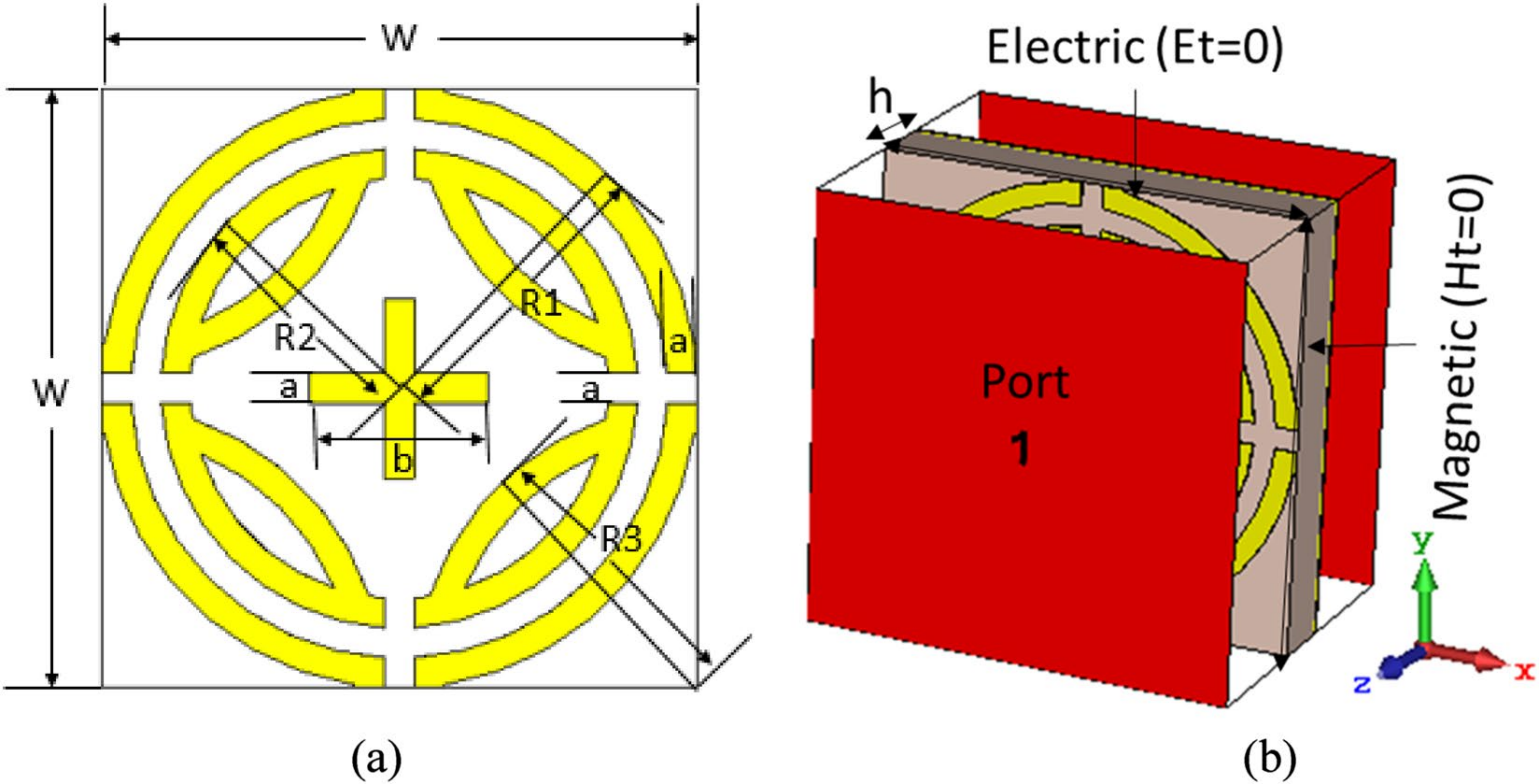
Design of proposed MPA (**a**) Front view, (**b**) Simulation setup (TEM). (CST STUDIO SUITE 2019, https://www.3ds.com/products-services/simulia/products/cst-studio-suite)^32^.

The proposed design consists of vertically and horizontally symmetrical split ring resonators with an inner cross, where the radius of the inner and outer ring is R2 and R1, respectively. Four crescent segments with radius R3 are incorporated with inner split rings. A cross structure is placed at the center of the inner ring. The inner cross contributes to achieve lower absorption. The FR-4 substrate material with dielectric constant 4.3, thickness 1.6 mm, and electrical tangent 0.025 is used as substrate. The ground is placed on the opposite side of the substrate, which blocks any transmission from the MPA. Copper material with thickness 0.035 mm and electrical conductivity of 5.96 × 107 S/m is used for patch and ground plane. The unit cell size is 10 × 10 × 0.16 mm^3^. In the simulation transverse electromagnetic mode (TEM), electric (Et = 0) and magnetic (Ht = 0) is applied along the x and y axes, respectively, and open (add space) is assigned along both the positive and negative z axes. Two waveguide ports are applied along the z direction. Figure 1b shows the simulation setup of proposed MMA (TEM mode). Unit cell boundary is assigned along the x and y axes for transverse electric (TE) and transverse magnetic (TM) model simulations. CST microwave studio based on finite integration technique is used for simulating the design, where the frequency-domain solver is used^32^. Default surface based tetrahedral meshing was chosen in the numerical study. The number of mesh cells were 69,218, with the cell per max model box edge was 10. The length of the shortest and longest mesh edge was 0.000004 and 4.347737, respectively. The minimum and maximum of all mesh cell quality values were 0.000007 and 0.997704, respectively. The smooth mesh equilibrate ratio was 1.5. Figure 1 shows the design of the proposed MPA.

**Figure 2:**
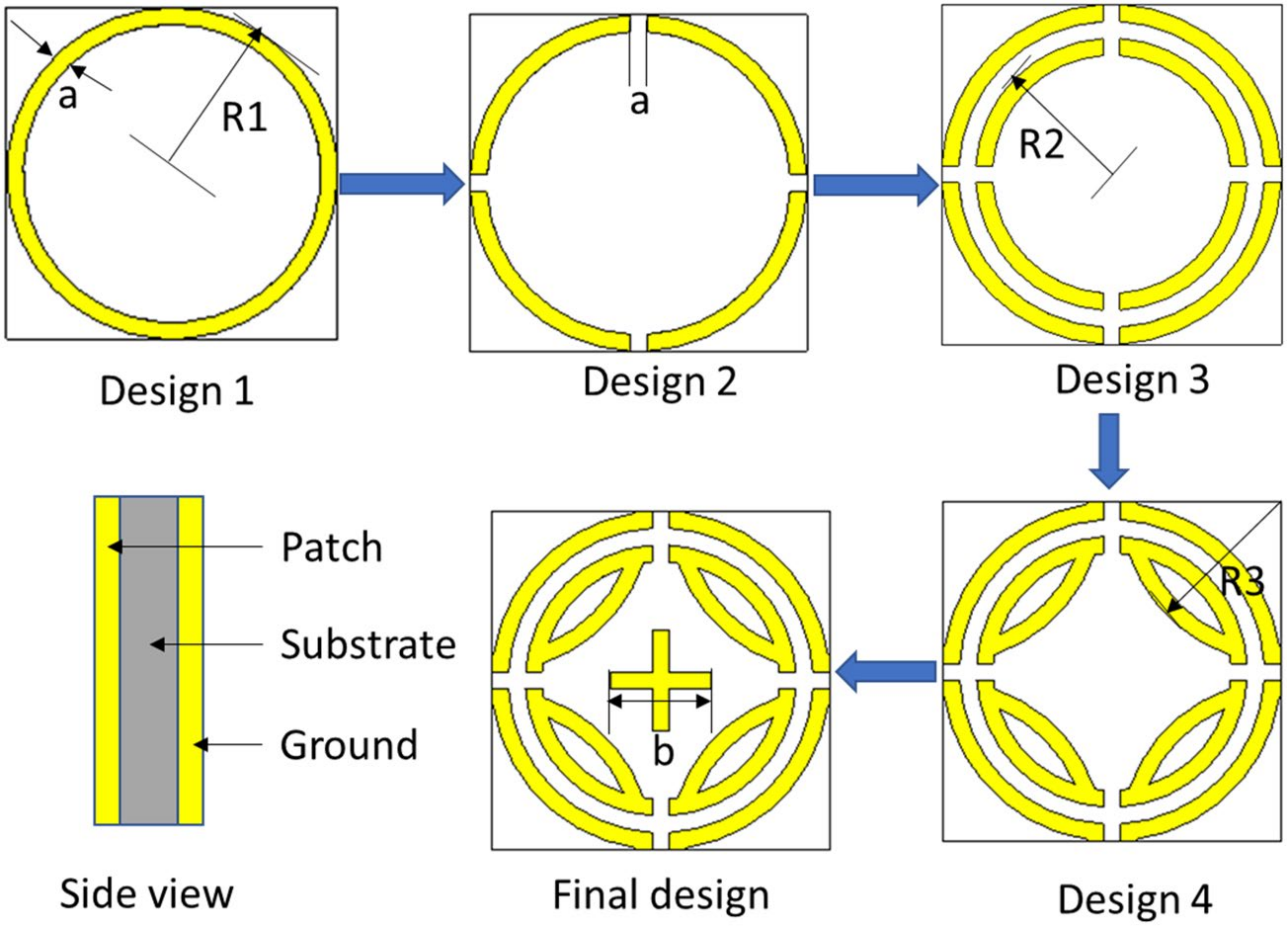
Design evaluation of proposed MPA. (CST STUDIO SUITE 2019, https://www.3ds.com/products-services/simulia/products/cst-studio-suite)^32^.

**Figure 3:**
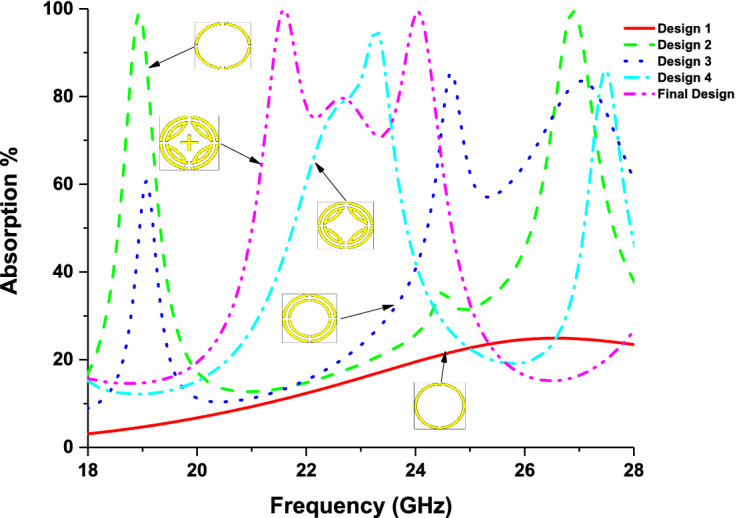
Absorption values at different design stages.

The design evaluation of the proposed MPA is illustrated in Fig. 2. Initially, a single ring is chosen in design 1 and no absorption peak is developed due to the poor capacitances formed between the single ring and the ground. Then, the ring is splinted into four equal segments (design 2) and two absorption peaks are found at 19 GHz and 27 GHz frequency due to additional capacitance created by splits. Coupling capacitance is created by placing another ring with four splits inside the outer ring, which makes a shift of absorption peaks at 24.25 GHz and 27 GHz frequency. Moreover, the inductance generated by the inner ring makes the absorption band wider. In design 4, four segments of the ring are connected with the inner ring to increase capacitance and inductance, which contributes to a shift absorption peak at 23 GHz. Finally, by adding a cross inside the inner ring, two perfect absorption bands are obtained at 21.6 GHz and 24.04 GHz, with a 70% wide absorption band of 3.2 GHz (21.24–24.44 GHz). The absorption characteristics for different designs is illustrated in Fig. 3 (The absorption calculation method is discussed in results and discussion section). The final design parameters are listed in Table 1.

**Table 1:** Design parameters of the proposed MPA.

Parameters	Value (mm)	Parameters	Value (mm)
W	10	R3	4.5
R1	5	a	0.5
R2	4	b	3

## Results and discussions

In this section the theory of absorption calculation from simulated results and its behavior of TEM, TE, and TM modes is discussed in subsection. 3.1 and 3.2. The metamaterial property and absorption behaviors for different (0°, 45°, and 90°) polarization angles, e-field, h-field, and surface current distributions, equivalent circuit model, and measurements are illustrated in 3.3–3.6.

### Absorption calculation and Interference theory

The absorption of the proposed design is calculated using Eq. 1^20^.

$$ A(\omega ) = 1 - \Gamma (\omega ) - T(\omega ) $$

1 $$ A(\omega ) = 1 - \left| {S_{11} } \right|^{2} - \left| {S_{21} } \right|^{2} $$

where $\Gamma (\omega ) = \left| {S_{11} } \right|^{2} =$ reflection coefficient and $T(\omega ) = \left| {S_{21} } \right|^{2} =$ the transmission coefficient obtained from simulation. In the proposed MMA a complete copper ground plane is on the back side, which has resistivity $\rho = 1.72\Omega$, permeability $\mu = 1$, and conductivity $\sigma = 5.8 \times 10^{7}$ S/m. The skin depth of the EM wave is calculated by $\delta = \sqrt {{\rho \mathord{\left/ {\vphantom {\rho {\pi f\mu }}} \right. \kern-\nulldelimiterspace} {\pi f\mu }}}$ at 21 GHz, $\delta = 0.005$ mm, where 0.035 mm thick copper ground plane is enough to restrict the transmission of the EM wave. Results as transmission coefficient (S_21_) become zero, as shown in Fig. 2(b). So, Eq. (1) becomes,

$$ A(\omega ) = 1 - \left| {S_{11} } \right|^{2} $$

**Figure 4:**
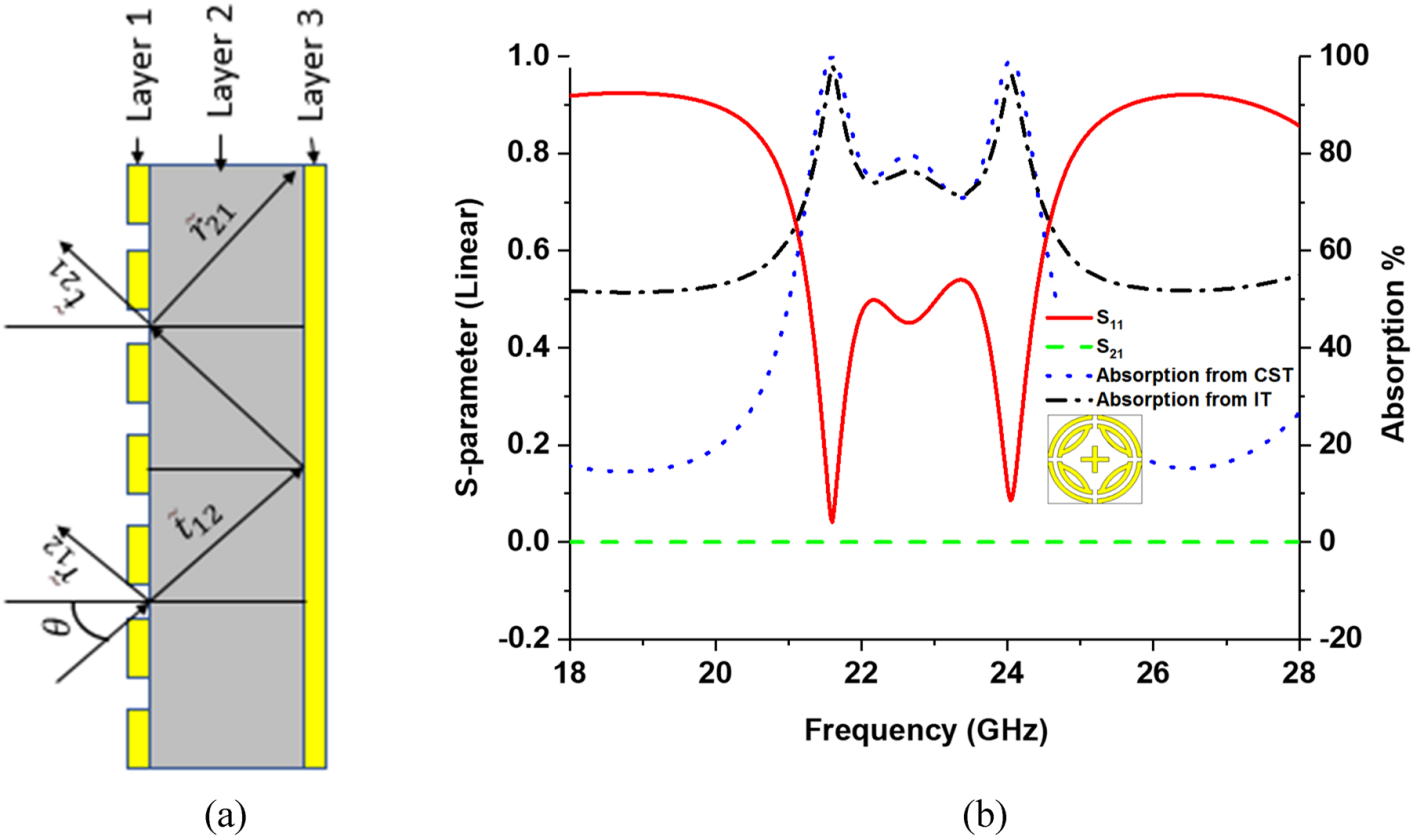
(**a**) Interference theory model, (**b**) S-parameters and absorption plot.

The absorption is also calculated by using interference theory (IT) model, shown in Fig. 2. The incident wave from the air-spacer on layer 1 is partially reflected back to the air with reflection coefficient $\tilde{r}_{12} = r_{12} e^{{j\phi_{12} }}$ and is partially transmitted to layer 2 with transmission coefficient $\tilde{t}_{12} = t_{12} e^{{j\theta_{12} }}$. The transmitted wave propagates to the ground with a complex propagation phase $\tilde{\beta } = \beta_{r} + i\beta_{i} = \sqrt {\varepsilon_{Substrate} } k_{0} d$, where $\beta_{r}$ is the propagation phase,$\beta_{i}$ is the absorption of substrate, $k_{0}$ = free space wave number, and $d$ = substrate thickness. Another propagation phase $\tilde{\beta }$ is added after a total reflection from the ground. The reflected wave is again partially transmitted to air-spacer with transmission coefficient $\tilde{t}_{21} = t_{21} e^{{j\theta_{21} }}$ and reflected with reflection coefficient $\tilde{r}_{21} = r_{21} e^{{j\phi_{21} }}$. The overall reflection is superimposed of multiple reflections of the model expressed in Eq. (2) ^33,34^ and absorption is calculated by using Eq. (3). Figure 4) shows the interference theory model and Fig. 4b shows S-parameters and absorption of the proposed design, where two perfect absorption picks are found at 21.6 and 24.04 GHz. The peak absorptive frequencies are obtained from the interference theory and keep consistent with the simulated result, though a slight difference is observed in non-operating regions. The possible reason for the slight frequency shifts could be attributed to the approximation of the complex wave number in the medium of the dielectric substrate^35^.

2 $$ \tilde{r} = \tilde{r}_{12} - \frac{{\tilde{t}_{12} \tilde{t}_{21} e^{{i2\tilde{\beta }}} }}{{1 + \tilde{r}_{21} e^{{i2\tilde{\beta }}} }} $$

3 $$ A(\omega ) = 1 - \tilde{r} $$

### Absorption analysis

**Figure 5:**
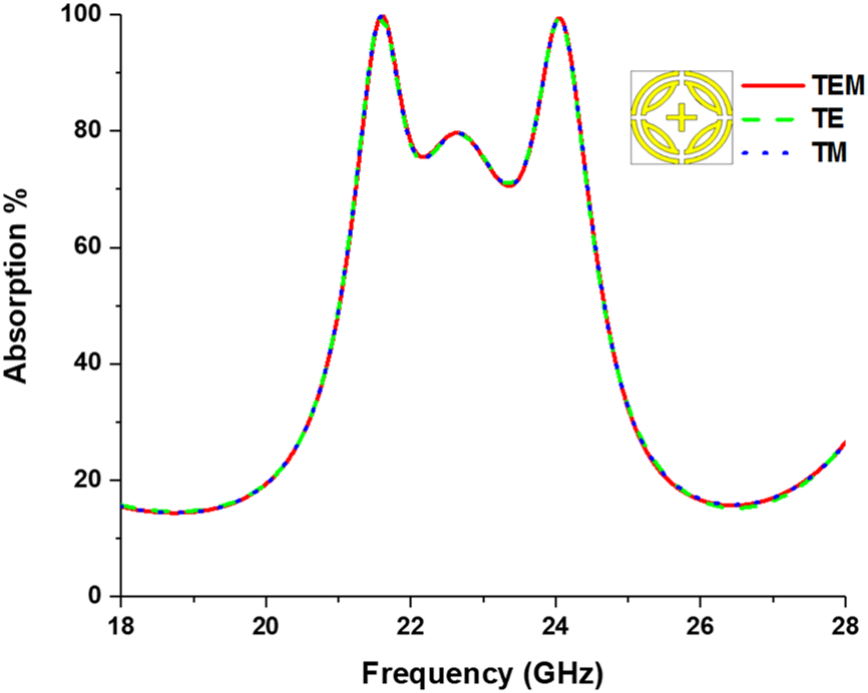
Absorption values for TEM, TE, and TM modes.

Proposed MPA is simulated at TEM, TE, and TM modes for validating its identical absorption behavior. Figure 5 shows the absorption, showing similar absorption characteristics for all modes due to vertical and horizontal symmetricity. Peak absorption of 99.9% appears at both 21.06 GHz and 24.04 GHz resonant frequency. Moreover, above 90% absorption bandwidth of 400 MHz and 760 MHz is achieved from 21.4–21.8 GHz and 23.84–24.24 GHz, respectively. In addition, above 70% absorption bandwidth of 3.2 GHz is achieved from 21.24–24.44 GHz frequency.

### Polarization incident angle analysis

**Figure 6:**
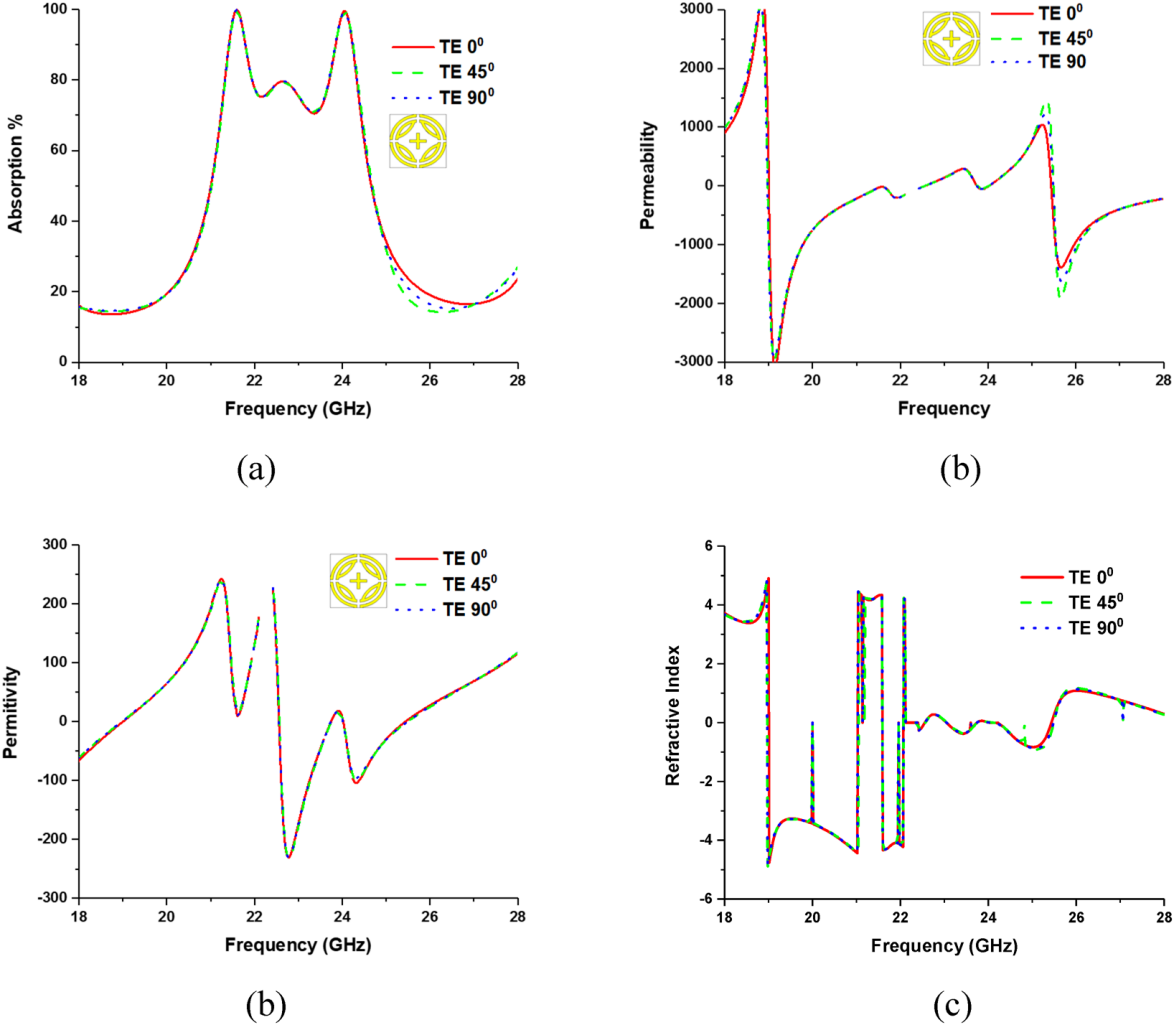
Metamaterial properties of the proposed MPA for different polarization angles at TE mode (**a**), permittivity, (**c**) permeability and (**d**) Refractive Index.

**Figure 7:**
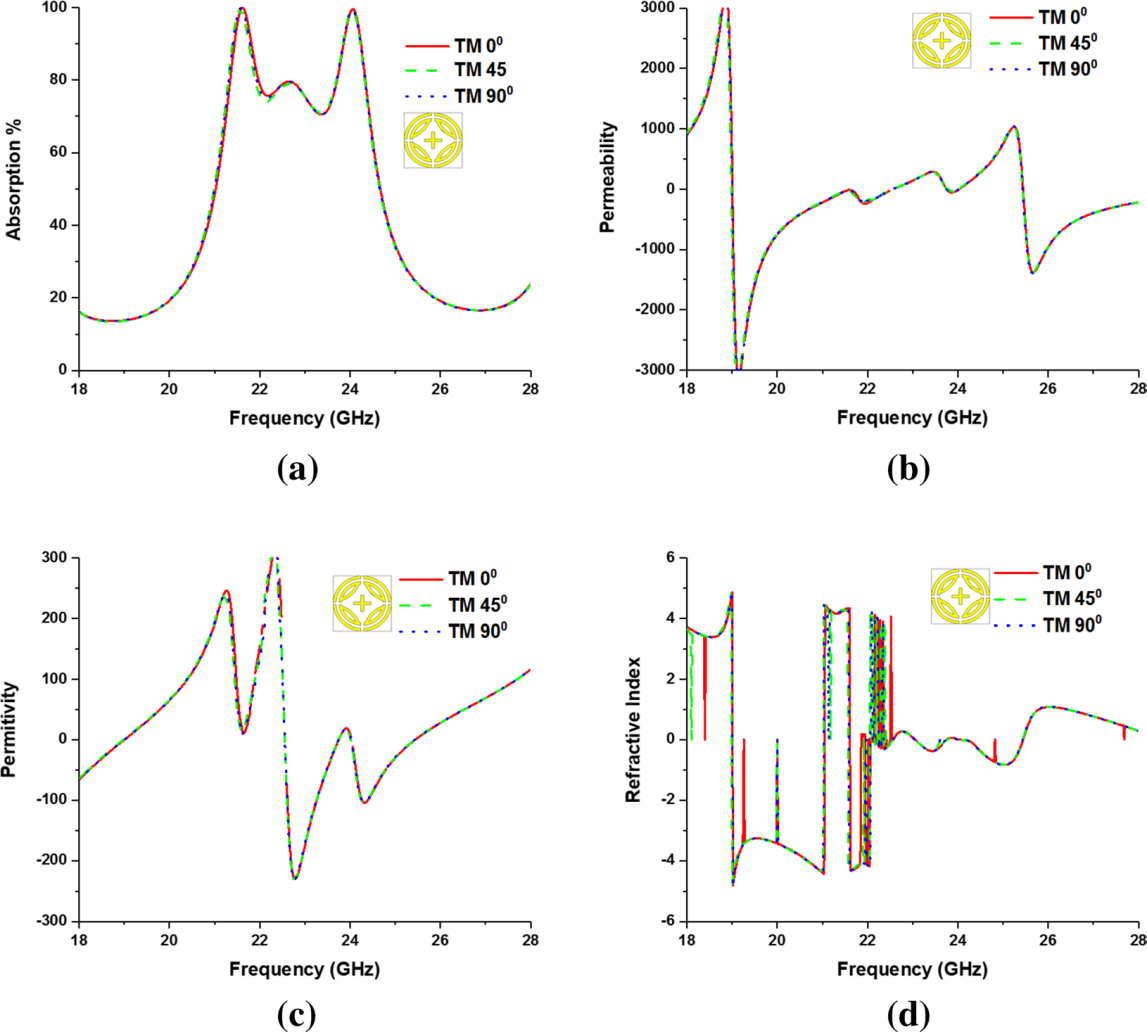
Metamaterial properties of the proposed MPA for different polarization angles at TM mode (**a**) absorption, (**b**) Permeability, (**c**) Permittivity, and (**d**) Refractive Index.

**Figure 8:**
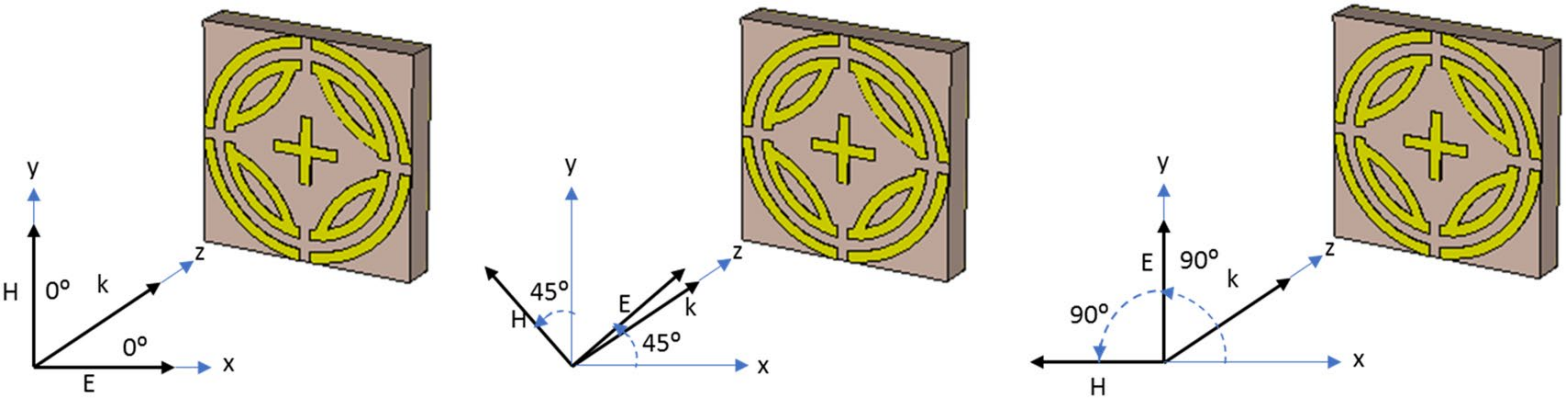
Polarization insensitivity of different incident angles. (CST STUDIO SUITE 2019, https://www.3ds.com/products-services/simulia/products/cst-studio-suite)^32^.

The metamaterial property and polarization insensitivity for 0°, 45°, and 90° are investigated for both TE and TM modes, shown in Figs. 6 and 7, respectively. In Fig. 8, propagation of wave vector k is in z direction, and H and E vectors are in y and x directions, respectively. Due to the axial and rotational symmetricity of proposed MMA the absorption behavior is polarization insensitive up to 90° incident angles^29^. Permittivity and permeability of proposed MPA are calculated by using Eqs. (4 and 5), respectively^30,36^:

4 $$ \varepsilon_{r} = \frac{2}{{\sqrt { - k_{\theta } d} }}\frac{{1 - v_{1} }}{{1 + v_{1} }} $$

5 $$ \mu_{r} = \frac{2}{{\sqrt { - k_{\theta } d} }}\frac{{1 - v_{2} }}{{1 + v_{2} }} $$

where $\varepsilon_{r} =$ permittivity, $\mu_{r} =$ permeability, $d =$ thickness of the substrate,$v_{1} = S_{21} + S_{11}$ ,$v_{2} = S_{21} - S_{11}$ and wave number,$k_{\theta } = {\omega \mathord{\left/ {\vphantom {\omega c}} \right. \kern-\nulldelimiterspace} c}$, $\omega = 2\pi f$($f$ = applied frequency as EM wave), and $c =$ velocity of light. Due to the copper ground, the transmission (S_21_) through the MMA is zero and Eq. 4 becomes $\varepsilon_{r} = \frac{2}{{jk_{\theta } d}}\frac{{1 - S_{11} }}{{1 + S_{11} }}$. Though the wave number ($k_{\theta }$ ), and substrate thickness are constant, complex value S_11_ plays a significant role in achieving negative permeability. The magnitude of S_11_ can be controlled by a varying number of rings, their gap, splits gap, and the dimension of the inner cross. Moreover, the variation of these parameters changes the inductance and capacitance of the resonator, which results in a change of S_11_ as well as that of the permittivity and permeability. Double negative property (DNG) is found at 24.04 GHz frequency band and single negative property is found at 21.6 GHz frequency band for both TE and TM modes. The refractive index of the proposed MPA can be calculated by Nicolson-Ross-Weir (Eq. 6) and Direct Refractive index (Eq. 7) methods, where Eq. 7 is used to calculate. The results show negative refractive index for both resonant frequencies in all polarization incident angles. Absorption and metamaterial parameters are tabulated in Table 2.

6 $$ \eta = \sqrt {\varepsilon_{r} \mu_{r} } $$

7 $$ \eta = real\left[ {\frac{c}{i\pi ft}\sqrt {\frac{{\left( {S_{21} - 1} \right)^{2} - (S_{11} )^{2} }}{{\left( {S_{21} - 1} \right)^{2} + (S_{11} )^{2} }}} } \right] $$

**Table 2:** Polarization incident angle and metamaterial properties of the proposed MA.

EM Wave mode	Polarization angle	Resonant frequency GHz	Absorption bandwidth	Permittivity	Permeability	Refractive Index	Maximum absorption
TE	0°	21.6	0.4	10.31	− 19.54	− 4.33	99.99%
		24.04	0.76	− 6.60	− 11.35	− 0.0034	
	45°	21.6	0.4	15.79	− 24.44	− 4.34	
		24.04	0.76	− 6.80	− 9.98	− 0.004	
	90°	21.6	0.4	11.52	− 21.12	− 4.33	
		24.04	0.76	− 10.38	− 5.75	− 0.0064	
TM	0°	21.6	0.4	21.9	− 11.40	− .4.33	99.99%
		24.04	0.76	− 11.88	− 15.30	− 0.005	
	45°	21.6	0.4	13.27	− 26.85	− 4.32	
		24.04	0.76	− 19.13	− 7.55	− 0.006	
	90°	21.6	0.4	9.9	− 20.03	− 4.33	
		24.04	0.76	− 17.05	− 9.87	− 0.006	

**Figure 9:**
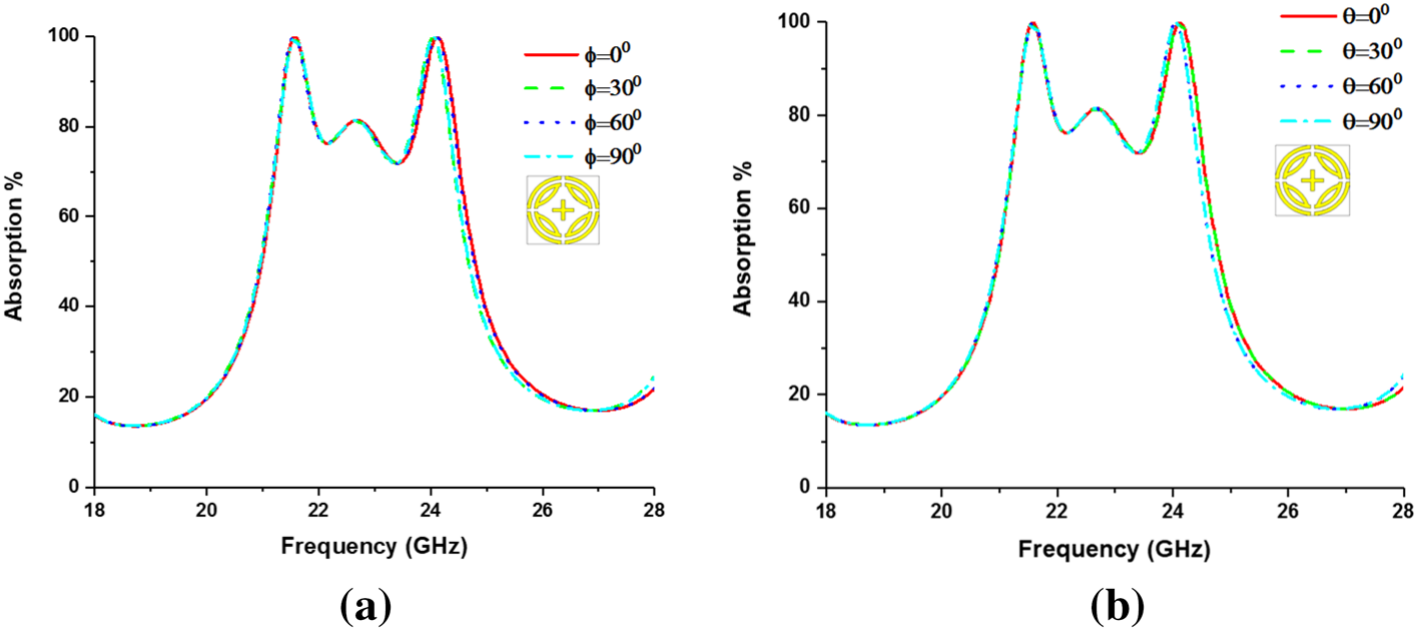
Absorption of the proposed MPA at (**a**) Normal, and (**b**) Oblique incident angles.

The incident angle insensitivity of the proposed MPA in TEM mode is also investigated, where the designed structure is found as incident angle insensitive due to its vertical and horizontal symmetricity. For both normal (ф) and oblique (θ) incidents of EM wave the MPA structure is capable of energy harvesting, absorbing, sensing, and many other applications. The identical absorption behavior for different incident angles of ф and θ is shown in Fig. 9.

### E-fields, H-fields and Surface current analysis

**Figure 10:**
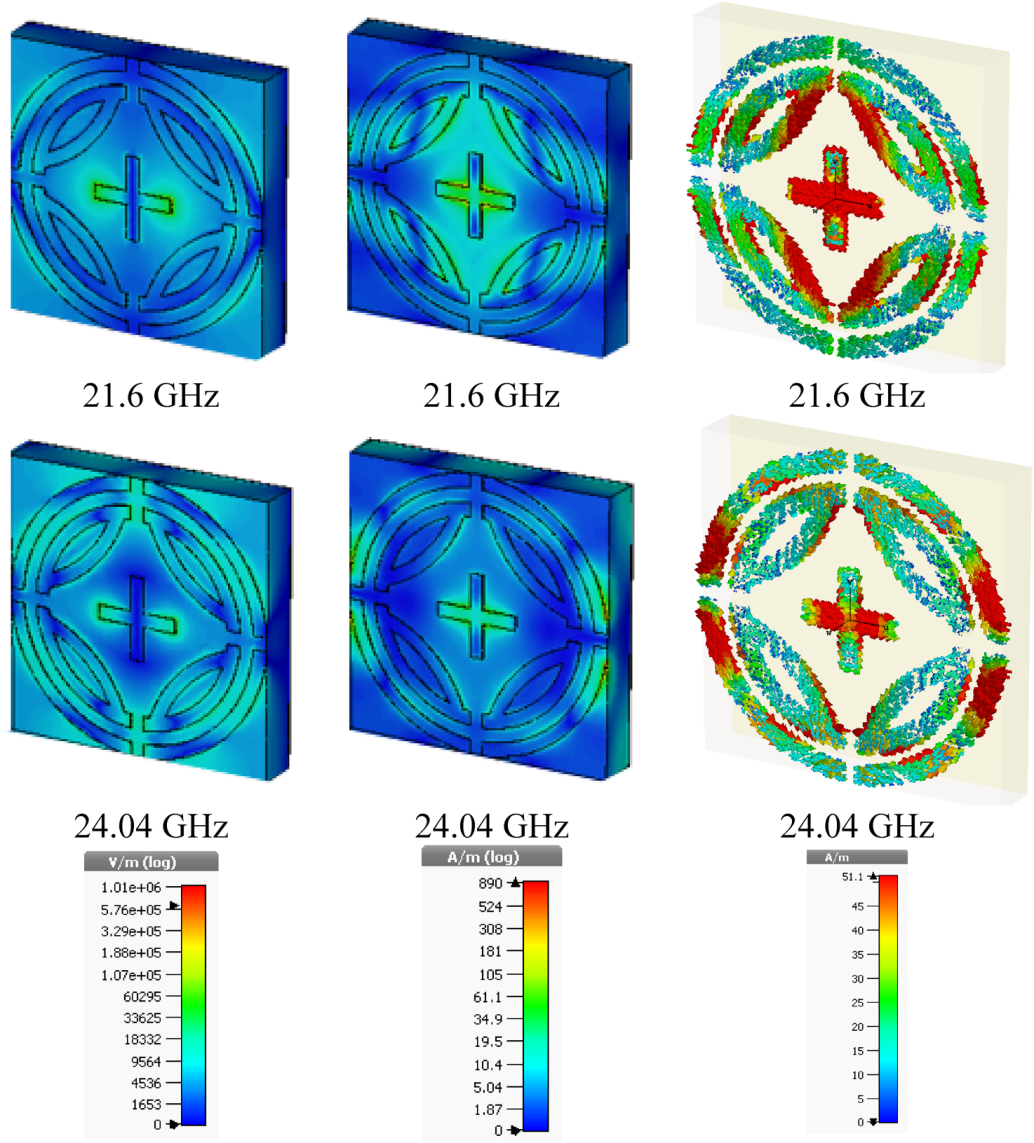
E-field, H-field, and surface current for TE mode. (CST STUDIO SUITE 2019, https://www.3ds.com/products-services/simulia/products/cst-studio-suite)^32^.

**Figure 11:**
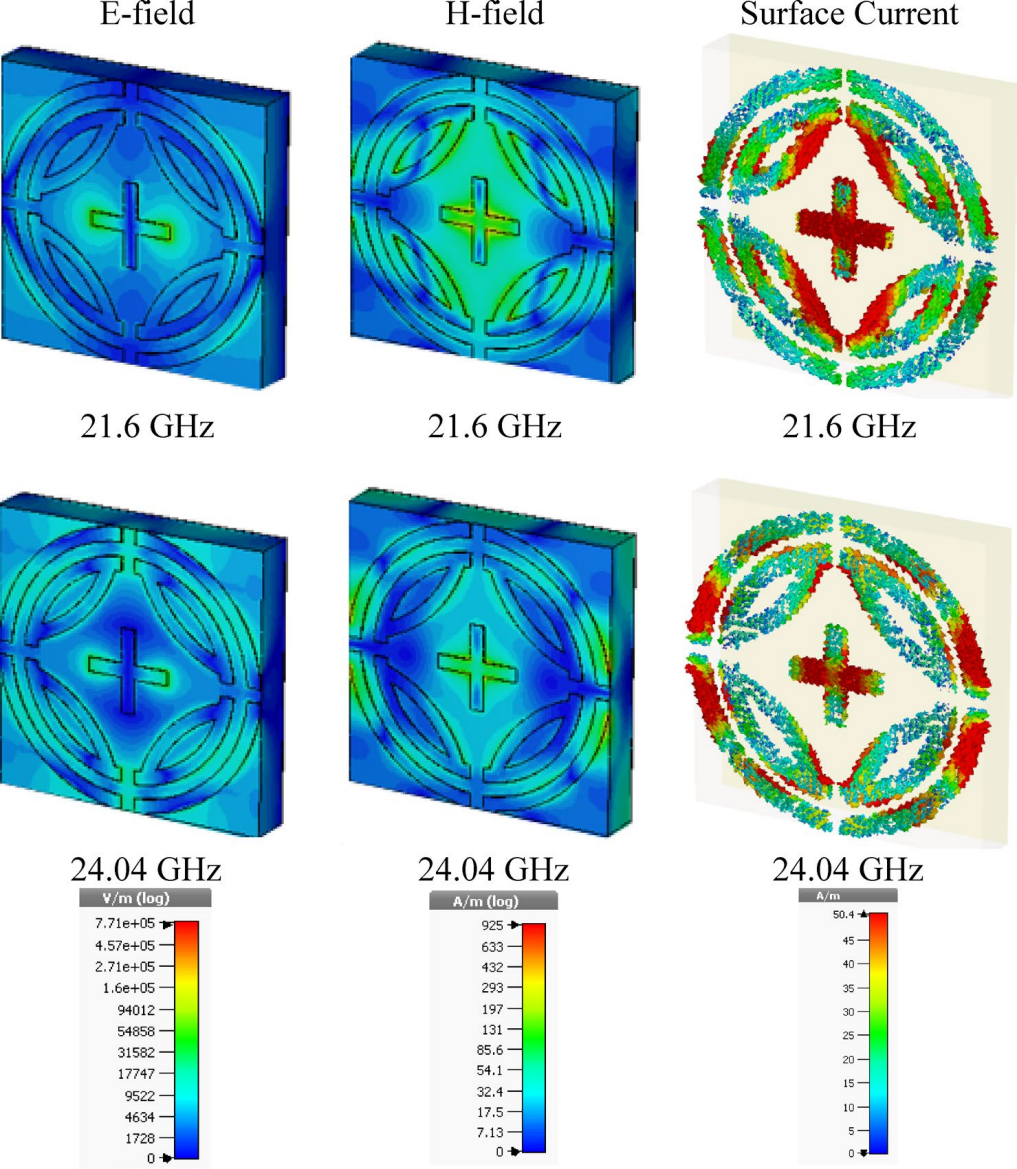
E-field, H-field, and surface current for TM mode. **(CST STUDIO SUITE 2019, **https://www.3ds.com/products-services/simulia/products/cst-studio-suite)^32^.

E-field, H-fields, and the surface distribution of proposed MPA are illustrated in Fig. 10 and Fig. 11 for TE and TM modes, respectively. The relation between these three can be understood by Maxwell's equation^25,30^, which relates magnetic field with the electric field and current distribution as given below,

8 $$ \nabla \times H = J + \in \frac{\partial E}{{\partial t}} $$

Also, the relation between electric field and current density is

9 $$ J = \sigma E $$

Figures 10 and 11 show that H-field is inversely changed along with the direction of E-field. The E-field is stronger at 24.04 GHz frequency than 21.6 GHz frequency, and thus negative permittivity is experience at 24.0 GHz frequency. However, a negative value of permeability reduces at 24.0 GHz frequency because h-field is less influential than 21.6 GHz frequency. For both TE and TM modes the similar E-field and H-field behaviors are found for both resonant frequencies, which is shown in Figs. 10 and 11, respectively.

### Equivalent circuit model

**Figure 12:**
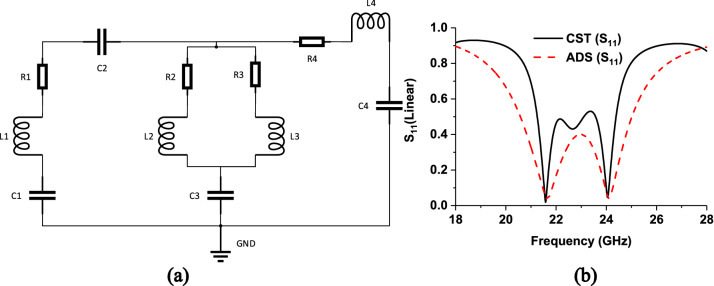
(**a**) Equivalent circuit design and simulation by ADS, where values are R1 = 47.70 Ω, L1 = 5.64 nH, C1 = 0.0098 pF, C2 = 0.015 pF, L2 = 8.48 nH, R2 = 8.37 Ohm,C3 = 0.023 pF, L3 = 4.43 nH, R3 = 6 Ohm, R4 = 5.92 Ohm, L4 = 2.91 nH, C4 = 0.0098 pF. (b) S_11_-parameters (linear) from ADS and CST. (PathWave Advance Design System (ADS), https://www.keysight.com/sg/en/lib/resources/software-releases/pathwave-ads-2019.html)^37^.

The equivalent circuit diagram of the proposed MPA is simulated by PathWave Advance Design System (ADS) software^37^ and presented in Fig. 12a. An RLC series circuit for outer split ring and RLC series–parallel combination for inner segment parallelly connected with capacitor C2. For inner cross-segment R4 and L4 are used, which relate to ground capacitor C4 in the series. S-parameters (linear) for both ADS and CST are presented in Fig. 12b, which shows identical patterns, though a small discrepancy is found.

### Experimental results

**Figure 13:**
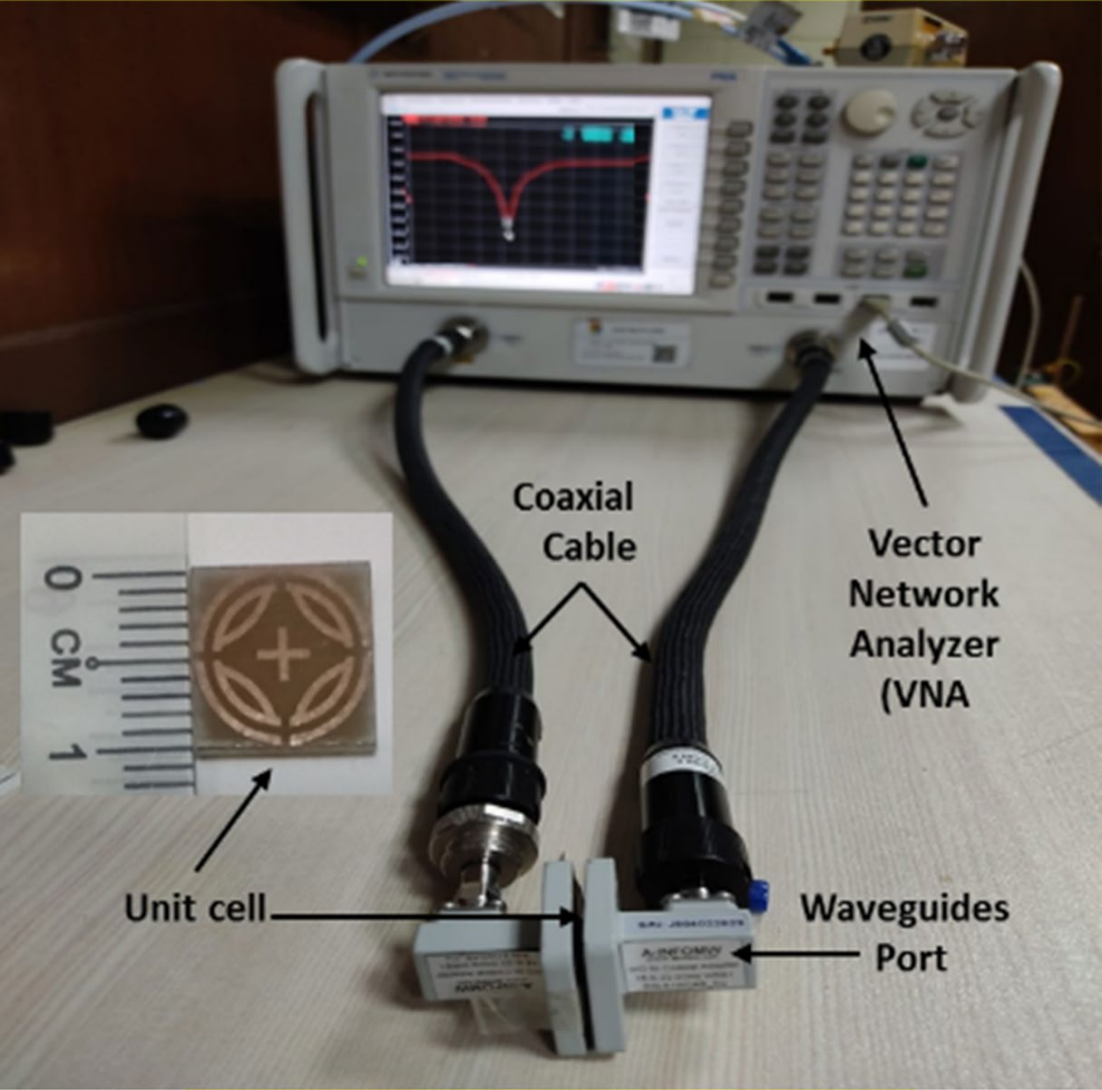
Unit cell measurement set-up.

**Figure 14:**
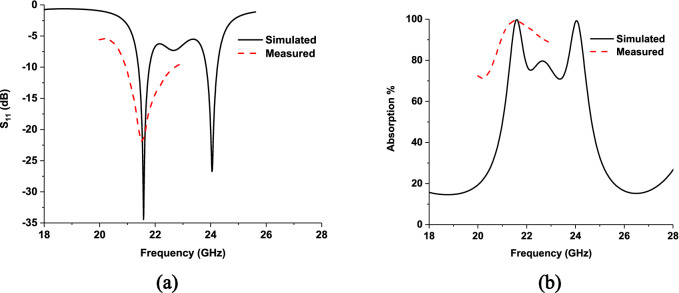
Simulated and measured result (**a**) S_11_ parameters, (**b**) absorption of the proposed MPA.

The unit cell measurement setup is presented in Fig. 13, where A-INFOMW WR51 waveguides are used to measure reflection and transmission coefficients. The upper cutoff frequency of the waveguide is 23.143 GHz. The measured S_11_ parameters and absorption are presented in Fig. 14, where 99.99% peak absorption is found at a 21.6 GHz lower frequency band. The second band could not be measured due to measurement limitations. Figure 14 shows that the measured result is consistent with simulated result.

## Sensor applications

**Figure 15:**
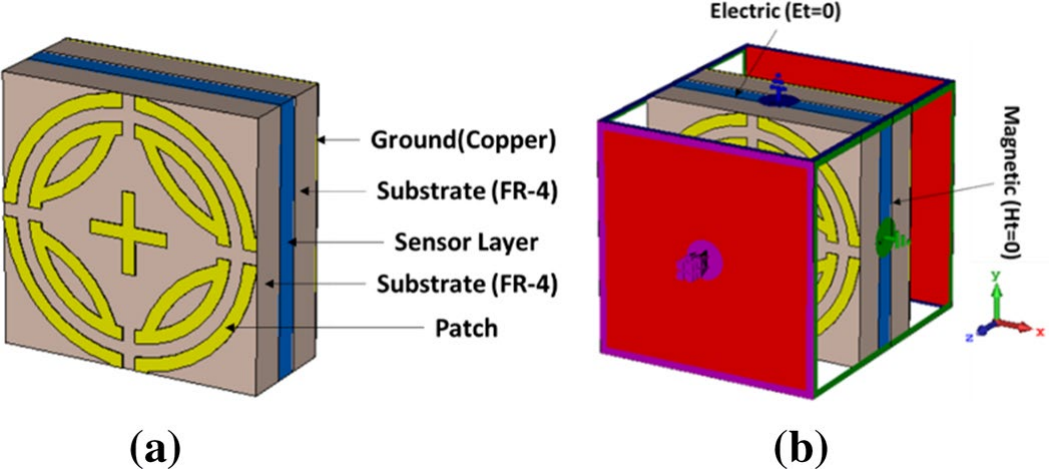
(**a**) Sensor model (**b**) Simulation setup. **(CST STUDIO SUITE 2019, **https://www.3ds.com/products-services/simulia/products/cst-studio-suite)^32^.

**Figure 16:**
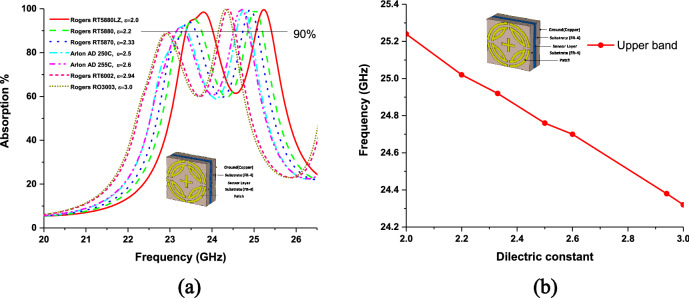
(**a**) Frequency vs absorption plot for different material (**b**) sensitivity for different dielectric constant.

**Figure 17:**
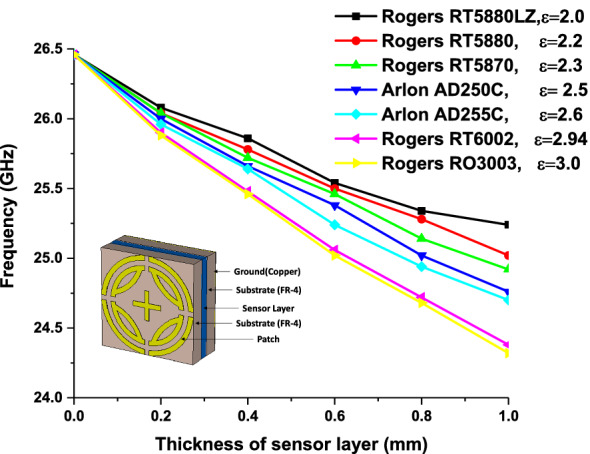
Sensitivity of the pressure sensor analysis for different thickness of sensor layer.

**Figure 18:**
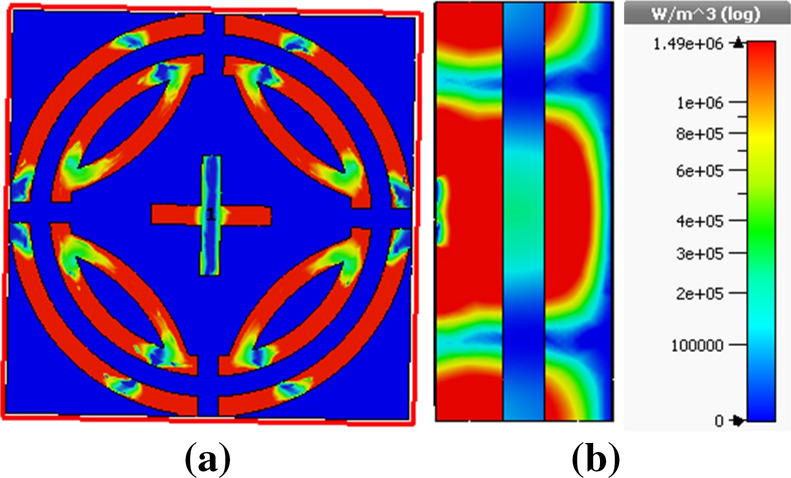
Power loss density of proposed sensor model (**a**) front view (**b**) side view. **(CST STUDIO SUITE 2019, **https://www.3ds.com/products-services/simulia/products/cst-studio-suite)^32^.

Metamaterial sensing has recently been investigated on a large scale^20^. Though this topic is of great interest to researchers, the sensing application of k band has been investigated using proposed MPA. Proposed MPA has identical absorption properties for polarization angles up to 90° with peak absorption of 99.99% at 240.4 GHz, which makes it an appropriate candidate for sensor application. A sensor model is presented in Fig. 15, where a 1 mm thick sensor layer is placed between two substrates^2^. Sensitivity for different dielectric constants and sensor layer thickness was investigated, as shown in Figs. 16 and 17, respectively. At first, the sensing performance was investigated by changing the dielectric constant of the sensing layer. This change had a significant impact on the capacitance of the microstrip line, which resulted in shifting at the resonance frequency of the MMA, as illustrated in Fig. 16. Figure 16a shows the absorption behavior of MMA is also changed for the variation of the dielectric constant. A strong sensitivity was found at both upper and lower bands. But sensing capability was estimated by the considered upper band because the absorption peaks at the upper band and remained at 99.9%. Moreover, the absorption at lower band decreased (below 90%) due to the sensor layer dielectric constant and change in overall thickness. These changes occurred due to the change in mutual and coupling capacitance of the patch^2^. Figure 16b shows that the absorption peak shifted towards the lower resonating frequency when the sensor layer dielectric constant increased, showing a linear relation between frequency and dielectric constant. Besides, the power loss density of the proposed sensor model is shown in Fig. 18, which shows that the power loss of the sensor layer was different from both side substrate layers due to the difference of its dielectric constant. Then, the pressure sensor performance was investigated by changing substrate layer thickness, as shown in Fig. 17. The simulation was performed by changing layer thickness 0 to 1 mm (0.2 mm apart) for different materials (dielectric constant 2.0–3.0). Figure 17 shows that the capacitance of the absorber patch increased accordingly when the thickness of the sensor layer increased, and the resonant frequency moved toward a lower frequency region. Based on^2,20,25^, it can be said that the proposed MPA has great potentiality for sensing applications, like permittivity and pressure sensors.

## Comparison

A detailed comparison of the proposed MAP with relevant work is illustrated in Table 3, with considered unit cell size, operating frequency band maximum absorption, and polarization angle insensitivity properties. The proposed absorber has a maximum 99.99% absorption peak at the K band frequency region. Polarization angle insensitivity is found to be up to 90° for both TE and TM modes, which is rear in the relevant research. So, this property proposes one as a better candidate for K band sensing and absorption applications.

**Table 3:** Comparison of proposed MPA with relevant works.

References	Patch type	Symmetricity	Substrate material	Unit cell size	Operating frequency	Resonant Frequency	Maximum absorption	Polarization angle insensitivity	Applications
^31^	Quarter SR with inner asterisk resonator	Yes	FR-4 4.4	6 × 6 × 0.635	K	19.4 19.8	99.98% 99.94%	θ ≤ 85°	Absorber
^30^	Spiral shape	Yes	FR4	10 × 10 × 1.578	Ku and K	15.3 17.04 20.06 21.3	99.95%	θ ≤ 80°	Absorber
^24^	Shorted stub CR	Yes	FR-4 4.6	8.86 × 8.86 × 0.4	Ku	17 18	99.9% 99.83	θ ≤ 70°	Absorber
^27^	CSR and CCSR	Yes	FR-4 4.35	15.4 × 15.4 × 1	X Ku	11.15 16.1	99.9%	θ ≤ 60°	Absorber
^29^	Modified CSRR	Yes	FR-4 4.3	9 × 9 × 1.58	X Ku K	11.23 14.18 17.37 19.18	99.13%	θ ≤ 90°	Absorber
^25^	Ring C shape	Yes	FR-4 4.6	20 × 20 × 1.575	Ku	13.78 15.3	99.6%	θ ≤ 60°	Absorber and sensor
Proposed	CSRR	Yes	FR-4 4.3	10 × 10 × 1.6	K	21.6 24.04	99.99%	θ ≤ 90°	Absorber and sensor

## Conclusions

This paper proposed a CSRR metamaterial perfect absorber for K band application, where 400 MHz and 760 GHz and perfect 90% absorption bandwidth was realized from 21.4–21.8 GHz and 23.84–24.24 GHz frequency bands. Identical absorption characteristics were realized for all TEM, TE, and TM modes. Polarization insensitivity was investigated for both TE and TM modes and found a unique absorption curve for polarization angles up to 90°. E-field, H-field, and surface current distribution is also explained. Evaluation of design simulated equivalent circuit and sensor performance was investigated. Finally, a comprehensive analysis is illustrated through the tabular form, which shows excellent candidature of proposed MPA in K band applications like absorbing, sensing, filtering, invisible clock, etc.
